# Notch Cooperates with Survivin to Maintain Stemness and to Stimulate Proliferation in Human Keratinocytes during Ageing

**DOI:** 10.3390/ijms161125948

**Published:** 2015-11-03

**Authors:** Elisabetta Palazzo, Paolo Morandi, Roberta Lotti, Annalisa Saltari, Francesca Truzzi, Sylvianne Schnebert, Marc Dumas, Alessandra Marconi, Carlo Pincelli

**Affiliations:** 1 Laboratory of Cutaneous Biology, Department of Surgical, Medical, Dental and Morphological Sciences, University of Modena and Reggio Emilia, via del Pozzo 71, Modena 41121, Italy; elisabetta.palazzo@gmail.com (E.P.); 60645@studenti.unimore.it (P.M.); roberta.lotti@unimore.it (R.L.); annalisa.saltari@unimore.it (A.S.); francesca.truzzi@unimore.it (F.T.); alessandra.marconi@unimore.it (A.M.); 2 LVMH Recherche, 185 Avenue de Verdun, Saint Jean de Braye 45800, France; sschnebert@research.lvmh-pc.com (S.S.); mdumas@research.lvmh-pc.com (M.D.)

**Keywords:** Notch1, survivin, keratinocytes, stem cells

## Abstract

The Notch signaling pathway orchestrates cell fate by either inducing cell differentiation or maintaining cells in an undifferentiated state. This study aims to evaluate Notch expression and function in normal human keratinocytes. Notch1 is expressed in all epidermal layers, though to a different degree of intensity, with a dramatic decrease during ageing. Notch1 intracellular domain (N1ICD) levels are decreased during transit from keratinocyte stem cells (KSC) to transit amplifying (TA) cells, mimicking survivin expression in samples from donors of all ages. Calcium markedly reduces N1ICD levels in keratinocytes. N1ICD overexpression induces the up-regulation of survivin and the down-regulation of keratin 10 and involucrin, while increasing the S phase of the cell cycle. On the other hand, Notch1 inhibition (DAPT) dose-dependently decreases survivin, stimulates differentiation, and reduces keratinocyte proliferation in samples from donors of all ages. Silencing Notch downgrades survivin and increases keratin 10. In addition, Notch1 inhibition decreases survivin levels and proliferation both in KSC and TA cells. Finally, while survivin overexpression decreases keratinocyte differentiation and increases N1ICD expression both in KSC and TA cells, silencing survivin results in N1ICD down-regulation and an increase in differentiation markers. These results suggest that the Notch1/survivin crosstalk contributes to the maintenance of stemness in human keratinocytes.

## 1. Introduction

Homeostasis of the adult epidermis is ensured by a delicate equilibrium of proliferation, differentiation, and apoptosis [1]. Such a fine balance is maintained by keratinocyte stem cells (KSC) that reside in the basal epidermal layer. KSC generate transit amplifying (TA) cells that in turn undergo a limited number of cell divisions before committing to terminal differentiation [2]. This process begins when keratinocytes lose their attachment to the basement membrane and exit the cell cycle, giving raise to differentiating layers that provide a protective barrier for the entire body. Once the keratinocytes enter the first suprabasal layers, they express keratins 1 (K1) and 10 (K10), while involucrin as well as other proteins are expressed when epidermal cells move to the granular layer [3]. Although a myriad of signaling pathways have been detected in the human epidermis [4], the precise molecular mechanisms responsible for inducing cell cycle arrest and terminal differentiation or apoptosis are not completely understood. In particular, little is known about the control of KSC fate decisions and the transition to TA cells [5].

The Notch gene family encodes four transmembrane receptors (Notch1-4) that are activated by ligand binding and proteolytic cleavage, with the release of the Notch IntraCellular Domain (NICD) [6]. Once activated, NICD transmigrates into the nucleus where it associates with the DNA-binding protein CSL, converting it from a repressor into an activator of transcription [7,8], thus resulting in the expression of different target genes. Notch signaling is complex and appears to be dependent on the tissue context, being involved in either cell proliferation or differentiation. Although Notch seems to be critical for epidermal differentiation [9], there are conflicting data [10], which require clarification.

Activation of Notch results in direct activation of survivin gene transcription through at least one RPB-Jκ site in the survivin promoter [11]. Survivin is a member of the Inhibitor of Apoptosis Proteins (IAP) family, and is expressed in a few adult normal tissues, including skin, where it identifies KSC [12] and regulates the cell cycle [13]. 

While the number of KSC seems to persist throughout life [14], stem cell functions decline with age [15], and a reduced expression of some stem cell markers has been reported [16]. On the other hand, little is known on the behavior of either Notch or survivin in human skin during ageing.

The aim of the present study was to investigate the role of Notch1 in the epidermis of human donors of different ages. We present evidence that Notch1 maintains stemness in human keratinocytes *via* a bi-directional cross-talk with survivin, independent of age.

## 2. Results and Discussion

### 2.1. Notch1 Decreases during Ageing and Differentiation in Human Keratinocytes

**Figure 1:**
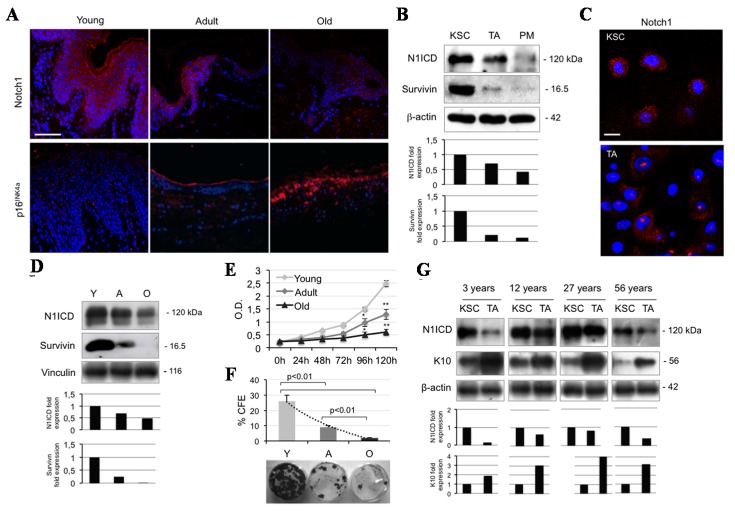
Notch1 levels decrease both during ageing and cell differentiation in human keratinocytes. (**A**) Immunofluorescence staining for Notch1 and p16^INK4a^ (red) in young, adult, and old skin biopsies. Cell nuclei were counterstained with DAPI (blue) (Bar = 200 µm); (**B**) Cells were analyzed immediately after separation, and levels of Notch1 activation (N1ICD) and survivin were determined by Western blot (WB) analysis. β-actin was used as loading control. Bar graphs show the average densitometry values normalized to β-actin; (**C**) Immunofluorescence staining was performed *in situ* on KSC (keratinocyte stem cells) and TA (transit amplifying) cell culture for survivin expression (red) and cell nuclei were counterstained with DAPI (blue) (Bar = 20 µm); (**D**) Survivin levels were analyzed immediately after isolation in young (Y), adult (A), and old (O) keratinocytes, and determined by WB analysis. Vinculin was used as loading control. Bar graphs show the average densitometry values normalized to vinculin; (**E**) The ability to proliferate *in vitro* of young, adult, and old keratinocytes was evaluated by MTT assay (* 0.01 < *p* < 0.05; ** *p* < 0.01); (**F**) Clonal growth assessment of young, adult, and old keratinocyte by CFE assay. At the bottom, representative pictures of CFE obtained by growing cells at clonal density and stained with CV are shown; (**G**) KSC and TA cells were analyzed immediately after separation, and levels of N1ICD and K10 (keratin 10) were determined by WB analysis. β-actin was used as loading control. Bar graphs show the average densitometry values normalized to β-actin.

Because Notch signaling has mostly been studied in the mouse system, scarce data are available on its expression in normal human keratinocytes. The present work shows that Notch1 is expressed in the basal layer, tends to decrease in the immediately suprabasal layers, and becomes more intense in the upper keratinocyte compartment (Figure 1A). There is a general agreement on the expression of Notch1 throughout all the epidermal layers [17,18], while *in situ* hybridization has nicely demonstrated that the highest level of transcription is in the basal layer of human interfollicular epidermis (IFE) [19,20]. Yet, there are some contradictory findings possibly related to studies performed in the hair follicle or in the mouse skin [21]. In order to definitely clarify Notch1 expression in IFE, we evaluated its levels in keratinocyte subpopulations, isolated according to their adhesive capacity to type IV collagen [22]. Notch1 intracellular domain (N1ICD) levels are higher in KSC than in TA cells, and almost absent in post-mitotic cells, mimicking survivin expression (Figure 1B). Confocal microscopy confirms the higher N1ICD expression in KSC (Figure 1C). We also demonstrate that Notch1 expression dramatically diminishes in sections from adult skin as compared to young specimens, while it almost disappears in old epidermis, displaying an irregular pattern of distribution. p16 INK4a, a marker of cell senescence, is absent in young skin, tends to increase in adult sections and it is strongly expressed in old skin (Figure 1A). Western blot analysis confirmed that N1ICD progressively decreases from young to old skin, and this is paralleled by a similar reduction in survivin levels in the same samples (Figure 1D). While the function of Notch1 in human epidermis is not clearly defined, we present evidence that keratinocytes from young subjects expressing high levels of Notch1 and survivin proliferate significantly more than adult or old keratinocytes, in a time-dependent manner (Figure 1E). Furthermore, keratinocytes from young samples generate the highest number of colonies, as compared to adult and old keratinocytes (Figure 1F). Finally, we wanted to evaluate N1ICD expression during transit between KSC and TA cells in samples from different age groups. First, it appears that Notch1 reduction during ageing is accounted for mostly by the lower levels of KSC in older keratinocytes, in keeping with the observation that Notch activation in the niche of germline stem cells is reduced with age, suggesting that Notch signaling regulates their niche occupancy [23]. Most importantly, we observed a constant reduction during differentiation from KSC to TA cells in all age groups. As control, K10 increases during transit from KSC to TA cells (Figure 1G). Taken together, these results demonstrate that Notch1 is strongly expressed in KSC of the IFE, and tends to decrease during differentiation and ageing.

### 2.2. Calcium Reduces N1ICD in Human Keratinocytes during Ageing

**Figure 2:**
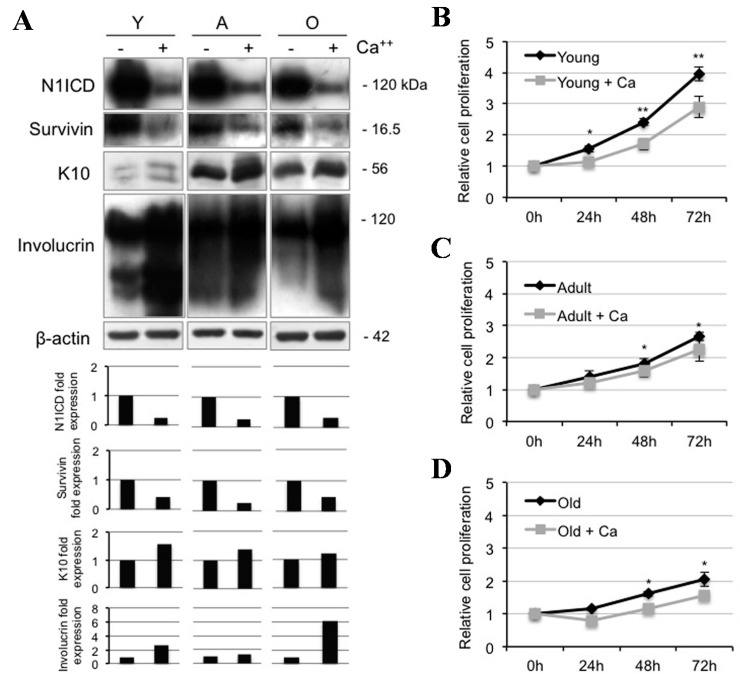
Calcium-induced differentiation decreases Notch1 activation during ageing. (**A**) Young (Y), adult (A), and old (O) keratinocytes were analyzed 24 h after 1.8 mM calcium treatment, and levels of Notch1 activation, survivin, K10, and involucrin were determined by Western Blotting analysis. β-actin was used as loading control. Bar graphs show the average densitometry values normalized to β-actin; (**B**) Relative cell proliferation of young keratinocytes with or without 1.8 mM calcium treatment by MTT assay; (**C**) Relative cell proliferation of adult keratinocytes with or without 1.8 mM calcium treatment by MTT assay; and (**D**) Relative cell proliferation of old keratinocytes with or without 1.8 mM calcium treatment by MTT assay. * 0.01 < *p* < 0.05; ** *p* < 0.01.

Notch signaling is involved in the regulation of cell fate. Depending on cell types and environments, Notch signaling induces cell differentiation or favors proliferation [24]. In order to shed light on the role of Notch1 in human epidermis, we provided keratinocytes from skin samples of different ages with a calcium ion. We first showed that calcium strikingly reduces N1ICD levels in samples from all age donors, consistent with the decrease of survivin in the same specimens, indicating that both Notch1 and survivin decrease during keratinocyte differentiation, in agreement with the previous finding of reduced Notch1 expression from KSC to TA cells (Figure 1B). As expected, K10 and involucrin levels increase upon addition of calcium (Figure 2A). Nickoloff and co-workers demonstrated that high calcium levels in co-presence with the JAG-1 peptide induced up-regulation of differentiation markers and stratification in the epidermal equivalent system [18]. In contrast to our data, they observed a slightly increased Notch expression when submerged cultures were raised to the air/liquid interface. Findings are difficult to compare, given that skin equivalents are characterized by an overall hyperproliferative pattern. While Nickoloff and colleagues failed to provide any proliferation experiments, we show here that calcium significantly reduces keratinocyte proliferation, particularly in young samples, in a time-dependent manner (Figure 2B–D). Finally, survivin has been previously detected in KSC, while it declines with keratinocyte differentiation both *in vivo* [25] and *in vitro*, with or without the addition of calcium [5].

### 2.3. Notch1 Inhibition Favors Differentiation and Reduces Proliferation in Young Human Keratinocytes

**Figure 3:**
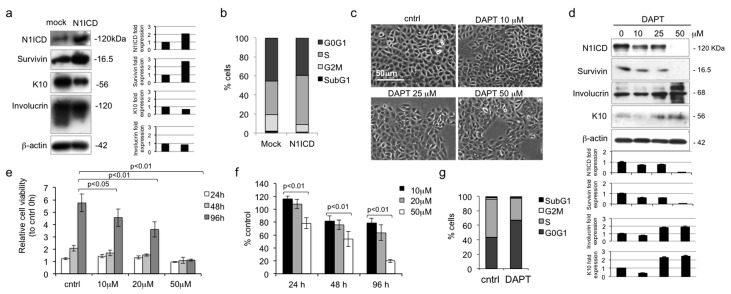
Notch1 inhibition reduces proliferation and increases differentiation in young keratinocytes. (**a**) Young keratinocytes were analyzed 48 h after pBABEpuro or pBABEpuro^N1ICD^ infection, and levels of N1ICD, survivin, K10 and involucrin were determined by Western blotting analysis. β-actin was used as loading control. Bar graphs show the average densitometry values normalized to β-actin; (**b**) Cell cycle was evaluated in young keratinocytes 48 h after pBABEpuro or pBABEpuro^N1ICD^ infection by flow cytometry; (**c**) Keratinocyte cultures of young donors were photographed at 24 h after 0, 10, 25, 50 µM DAPT treatment; (**d**) Cells were analyzed 24 h after DAPT treatment, and levels of N1ICD, survivin, involucrin, and K10 were determined by Western blotting analysis. β-actin was used as loading control. Bar graphs show the average densitometry values normalized to β-actin; (**e**) Relative ratio of young cell proliferation by MTT assay. Normalization was calculated as fold change compared to control at 0 h; (**f**) Relative percentile quantification of young cell proliferation by MTT assay. Normalization was calculated compared to 0 µM DAPT; (**g**) Cell cycle was evaluated in young keratinocytes 24 h after 50 µM DAPT treatment by flow cytometry.

To further understand the role of Notch, young human keratinocytes were infected with N1ICD cDNA. N1ICD overexpression induced the up-regulation of survivin and the down-regulation of K10 and involucrin (Figure 3A). Moreover, N1ICD overexpression markedly increased the S phase of the cell cycle in comparison with mock infected cells (Figure 3B), suggesting a role of Notch1 in keratinocyte proliferation. To confirm the effects of Notch1 in human keratinocytes, we first used an inhibitory approach, by using the γ-secretase inhibitor DAPT (0–50 µM). DAPT dose-dependently inhibits the achievement of keratinocyte confluency (Figure 3C). In addition, increasing doses of DAPT down-regulate survivin and up-regulate K10 and involucrin (Figure 3D), while reducing keratinocyte proliferation. In particular, at 48 and 96 h, we observed a significant decrease in keratinocyte proliferation in a dose-dependent manner (Figure 3E). When measured as percentage of control, increasing doses of DAPT significantly diminish keratinocyte proliferation at all time points (Figure 3F). Finally, DAPT markedly reduces S phase of the cell cycle (Figure 3G). Hence, these data suggest that Notch1 inversely correlates with differentiation and stimulates keratinocyte proliferation. While Notch signaling differs dramatically between cell types and according to various tissue contexts, most reports seem to suggest that Notch activity is associated with differentiation in both the IFE and the hair follicle [21]. On the other hand, it should be taken into consideration that most functional studies on Notch have been carried out in the mouse system [9], in particular in the hair follicle [26], which cannot be fully extrapolated for human skin. Indeed, it has been recently shown that Notch signaling enhances proliferation of epidermal stem cells by targeting Hes-1 [27]. Finally, this is consistent with the predominant expression of Notch1 in KSC (Figure 1B).

### 2.4. Notch1 Inhibition Reduces Keratinocyte Proliferation in Adult and Old Keratinocytes

**Figure 4:**
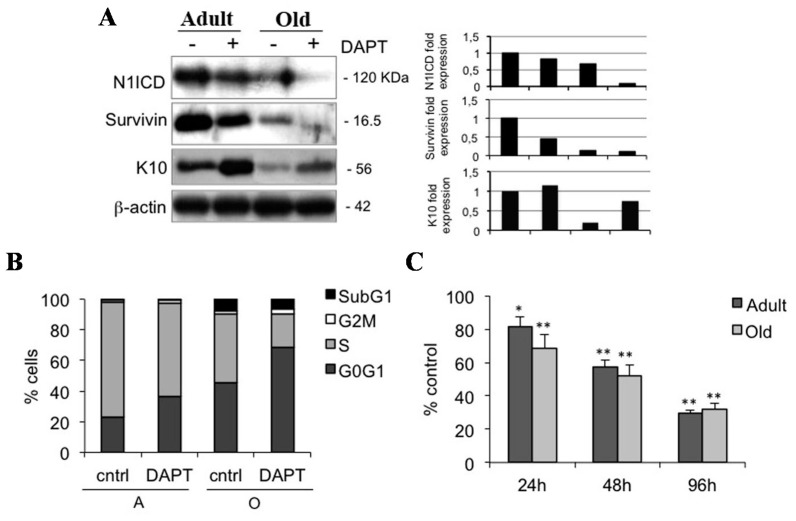
Notch1 inhibition decreases proliferation in adult and old keratinocytes. (**A**) Adult and old cells were analyzed 24 h after 0 and 50 µM DAPT treatment, and levels of N1ICD, survivin, and K10 were determined by Western blotting analysis. β-actin was used as loading control. Bar graphs show the average densitometry values normalized to β-actin; (**B**) Cell cycle was evaluated in adult and old keratinocytes 24 h after 0 and 50 µM DAPT treatment by flow cytometry; (**C**) Relative percentile quantification of adult and old cell proliferation after 50 µM DAPT treatment by MTT assay. Normalization was calculated as compared to control. * 0.01 < *p* < 0.05; ** *p* < 0.01.

To confirm the role of Notch1 in maintaining cells in an undifferentiated state, we also evaluated the effects of DAPT in keratinocytes from adult and old samples. Similar to what was observed in young keratinocytes, inhibition of Notch1 induces a down-regulation of survivin and an up-regulation of K10 in older specimens (Figure 4A). In addition, DAPT reduces the number of cells in S phase (Figure 4B), and decreases keratinocyte proliferation in samples from adult and old individuals, in a time-dependent manner (Figure 4C). These data confirm that Notch1 also favors keratinocyte proliferation in keratinocytes from older samples, and indicates that a reduced number of cells in S phase in old keratinocytes is consistent with a greater reduction of N1ICD levels in old relative to adult keratinocytes. This suggests that decreased proliferation in aged keratinocytes is associated with the lowest levels of N1ICD.

### 2.5. Notch1 Inhibition Down-Regulates Survivin and Inhibits Proliferation in Keratinocyte Subpopulations

**Figure 5:**
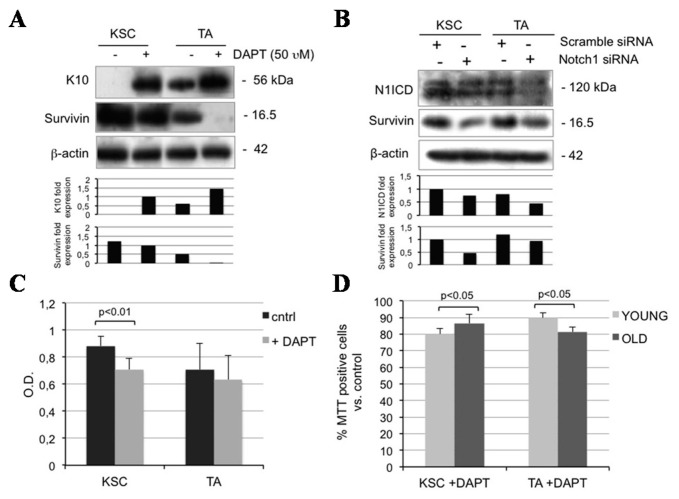
Notch1 inhibition affects both KSC and TA cells. (**A**) KSC and TA cells were analyzed 24 h after 0 and 50 µM DAPT treatment, and levels of survivin and K10 were determined by Western blotting. β-actin was used as loading control. Bar graphs show the average densitometry values normalized to β-actin; (**B**) KSC and TA cells were analyzed 48 h after Notch1 silencing, and levels of N1ICD and survivin were determined by Western blotting. β-actin was used as loading control. Bar graphs show the average densitometry values normalized to β-actin; (**C**) The ability to proliferate *in vitro* of KSC and TA cells after 0 and 50 µM DAPT treatment was evaluated by MTT assay; (**D**) Relative percentile quantification of both young and old KSC and TA cell proliferation after 50 µM DAPT treatment by MTT assay. Normalization was calculated compared to control.

Notch1 is mostly detected in KSC and seems to parallel the expression of survivin in the present work. Survivin regulates the cell cycle and has been reported to identify KSC [12]. Moreover, a functional Notch-survivin axis has been postulated in cancer [28]. To better understand the role of Notch in keratinocyte subpopulations in relation to survivin, we provided KSC and TA cells with DAPT and found that inhibiting Notch1 up-regulates K10 in both KSC and TA cells, down-regulates survivin levels in KSC, and to a greater extent, in TA cells (Figure 5A). Although DAPT is widely used as a tool to inhibit the Notch1 pathway, its activity is not specifically directed against Notch1, as it works on gamma secretase, the protein responsible for the activation of the receptor. Thus, to better understand the relationship between Notch and survivin, we silenced Notch1 via a siRNA and found a clear survivin down-regulation in both KSC and TA cells (Figure 5B). Moreover, DAPT significantly reduces proliferation in keratinocyte subpopulations from both young and old samples (Figure 5C,D). The complexity of the Notch signal is partially due to the high number of combined associations and to the regulation of down-stream target genes. Notch1 signaling induces survivin expression in lung cancer [29], while Notch1 and survivin co-segregate in breast cancer, where silencing Notch reduces survivin levels [28]. We hypothesize that the Notch/survivin axis is operational in normal human skin, resulting in keratinocyte proliferation and increased viability.

### 2.6. Survivin Up-Regulates Notch1 and Reduces Keratinocyte Differentiation

**Figure 6:**
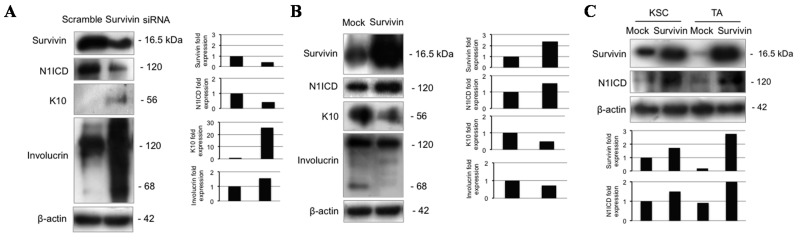
Survivin modulates Notch1 and keratinocyte differentiation. (**A**) Keratinocytes were analyzed 48 h after survivin silencing, and levels of survivin, N1ICD, K10 and involucrin were determined by Western blotting. β-actin was used as loading control. Bar graphs show the average densitometry values normalized to β-actin; (**B**) Keratinocytes were analyzed 48 h after pcz-CFG5.1-EGFP or pcz-CFG5.1-Survivin-EGFP infection, and levels of survivin, N1ICD, K10, and involucrin were determined by Western blotting analysis. β-actin was used as loading control. Bar graphs show the average densitometry values normalized to β-actin; (**C**) KSC and TA cells were analyzed 48 h after pcz-CFG5.1-EGFP or pcz-CFG5.1-Survivin-EGFP infection, and levels of survivin and N1ICD were determined by Western blotting analysis. β-actin was used as loading control. Bar graphs show the average densitometry values normalized to β-actin.

To further clarify whether the Notch-survivin axis described in cancer [30] also operates in normal human epidermis, we infected total keratinocytes with survivin siRNA, resulting in a marked Notch1 down-regulation, associated with K10 and involucrin up-regulation (Figure 6A). On the other hand, survivin overexpression induces Notch1 up-regulation, K10, and involucrin down-regulation (Figure 6B). Finally, survivin overexpression increases N1ICD both in KSC and TA cells (Figure 6C). These results indicate a bidirectional crosstalk between Notch and survivin in human epidermis, consistent with previous observations in breast cancer [28,30].

## 3. Experimental Section

### 3.1. Isolation of Primary Keratinocytes

Normal human keratinocytes were isolated from healthy skin biopsies obtained from waste materials from the Operating Room and were subdivided into three age classes of donors. The youngest age group ranged from 0 to 20 years (eight samples); the adult group aged between 21 and 49 (five samples); the oldest group was over 50 years (four samples). Patient consent for experiments was not required because Italian law considers human tissue left over from surgery as discarded material, as indicated from Modena Commission of Ethics. Isolated cells were cultured as described by Pincelli and colleagues [31].

Fresh keratinocytes were also divided into three populations and plated onto plastic dishes, coated for 2 h at 37 °C with type IV collagen 100 μg/mL (Sigma, St. Louis, MO, USA). They were first allowed to adhere to type IV collagen for 5 min (KSC-enriched), and the non-adherent cells were then transferred to fresh collagen-coated dishes and allowed to attach overnight (TA). Finally, keratinocytes not yet attached after one night were the third population (PM). The three keratinocyte populations were characterized on the basis of β_1_-integrin levels and colony-forming efficiency, as previously described [22]. Keratinocytes were immediately analyzed or cultured in serum-free Keratinocyte Growth Medium (KGM) (Lonza, Basel, Switzerland) and used for further experiments.

### 3.2. MTT Assay

Freshly isolated keratinocyte subpopulations were plated in a 96-well tissue culture plate (5000 cells/well), and MTT (3-(4,5-dimethylthiazol-2-yl)-2,5-diphenyltetrazolium bromide) (Sigma-Aldrich, St. Louis, MO, USA) assay was performed up to 120 h after plating. The results are expressed as the optical density mean ± SD of three independent experiments for each group of different ages.

For differentiation experiments, 1.8 mM calcium or diluent alone were added to the culture medium 24 h after plating. The MTT assay was performed up to 72 h after treatment. The OD values were converted to obtain relative cell proliferation as compared to cell culture at 24 h after seeding (0 h).

Finally, cell cultures were treated with different doses of DAPT (0, 10, 25 and 50 mM) 24 h after plating and MTT assay was performed up to 96 h after treatment. The results were converted to the percentage of non-treated cells or to relative cell viability, as compared to control at 24 h after seeding (0 h). The results are expressed as the mean ± SD of three independent experiments for each group of different ages.

### 3.3. siRNA Transfection of Keratinocytes

About 7 × 10^4^ cells/well were plated onto six-well plates in antibiotic-free KGM medium. 24 h later, cells were transfected twice with 25 nM scrambled or Notch1 siRNA (ON-TARGET plus siRNA code L007771, Dharmacon Inc., Lafayette, CO, USA) or Survivin siRNA (ON-TARGET plus SMARTpool code L003459, Dharmacon Inc), combined with Lipofectamin 2000 and Opti-MEM (Thermo Fisher, Waltham, MA, USA), as the datasheet suggests. 48 h after transfection, cells were lysed for WB analysis.

### 3.4. Infection of Keratinocytes

Total keratinocytes, KSC, or TA cells were plated in KGM, and 24 h later, infected twice with antibiotic-free conditioned medium containing pcz-CFG5.1-Enhanced Green Fluorescent Protein (EGFP) or pcz-CFG5.1-Survivin-EGFP retroviral vector (a kind gift from Achim Temme, Technische Universitat Dresden, Dresden, Germany) or empty pBABEpuro or pBABEpuro^N1ICD^ (kindly provided by Paolo Dotto, University of Lausanne, Lausanne, Switzerland) and/or polybrene to a final concentration of 0.8 μg/mL. Cells were lysed for WB analysis 48 h after infection.

### 3.5. Colony Forming Efficiency (CFE)

Keratinocytes were cultured on a feeder layer composed of mytomicin C (Sigma-Aldrich, St. Louis, MO, USA)-treated 3T3 cells at a density of 100 cells per dish. Fourteen days later, dishes were fixed with 10% buffered formalin and stained with crystal violet. Colonies were manually scored. The results were expressed as percentages of the number of cells plated in each dish and calculated as the mean ± SD of three independent experiments.

### 3.6. Flow Cytometry Analysis (FACS)

For cell cycle analysis, cells were fixed and permeabilized by Cytofix/Cytoperm Buffer (Becton Dickinson Biosciences, Franklin Lakes, NJ, USA). After 40 min incubation with DNAase at 37 °C, cells were stained with FITC-conjugated anti-BrdU monoclonal antibody. 7-aminoactinomycin (7-AAD) was added to each sample right before flow cytometry analysis (Epics XL flow cytometer, Beckman Coulter, Fullerton, CA, USA). The results are expressed as the percentage of cells in each phase of the cell cycle.

### 3.7. Western Blotting (WB)

Total proteins from cultured or fresh keratinocytes were extracted with RIPA lysis buffer containing protease inhibitors. Equal amounts of protein from each sample were run through a 6%–18% SDS-PAGE gel and transferred onto a nitrocellulose membrane. Briefly, membranes were incubated overnight at 4 °C with the following primary antibodies: rabbit polyclonal anti-human Notch1 (1:500; Abcam, Cambridge, UK), rabbit polyclonal anti-human survivin (1:1000; Novus Biologicals, Littleton, CO, USA), mouse monoclonal anti-human involucrin (1:1500; Sigma-Aldrich), rabbit polyclonal anti-human Keratin 10 (1:5000; Epitomics Burlingame, CA, USA) mouse monoclonal anti-human β-actin (1:5000; Sigma-Aldrich), or mouse monoclonal anti-human vinculin (1:400, Sigma-Aldrich). After three washes with a PBS/tween solution, membranes were then incubated with secondary antibodies: goat anti-mouse or goat anti-rabbit (1:3000; Bio-Rad Laboratories, Hercules, CA, USA) for 45 min at room temperature. Bands were then visualized with a chemiluminescence detection system (Amersham Biosciences UK Limited, Little Chalfont Buckinghamshire, UK). The band intensity was quantitatively determined using ImageJ software (Wayne Rasband, National Institute of Mental Health, Bethesda, MD, USA), and protein levels’ intensity was normalized to β-actin expression.

### 3.8. Immunofluorescence (IF) in Situ

KSC and TA cells were plated on chamber slides and, 48 h after seeding, were washed in PBS buffer and fixed *in situ* in 4% paraformaldehyde for 20 min and air dried. After a rehydration in PBS, cells were permeabilized for 10 min with 0.5% Tryton X-100 in 0.1% sodium citrate, treated for 5 min with 50 mM NH4Cl and incubated with 1% bovine serum albumin for 20 min. Slides were then incubated for 4 h at room temperature with the rabbit polyclonal anti-Notch1 antibody (1:50, Abcam). Then cells were incubated with secondary antibody, Alexa Fluor 546 (1:100, Thermo Fisher). Finally, cell nuclei were stained with 1 μg/mL Dapi (Sigma-Aldrich). Micrographs were taken on a Confocal Scanning Laser Microscope (Leica TCS SP2, Leica, Exton, PA, USA).

### 3.9. Immunofluorescence of Skin Biopsies

Skin sections (4 µm) from formalin fixed-paraffin biopsies were rehydrated in PBS buffer and permeabilized by incubation for 10 min with 0.5% Triton X-100. Then slides were incubated for 15 min with 0.5% bovine serum albumin and 5% goat serum, and for 60 min at 37 °C with the rabbit polyclonal anti-human Notch1 antibody (1:50, Abcam) or rabbit polyclonal anti-p16INK4a antibody (1:100, Abcam). After four washes in PBS, samples were incubated for 60 min with the anti-rabbit secondary antibody, Alexa Fluor 546 (1:100, Thermo Fisher Scientific). Fluorescent specimens were analyzed by a confocal scanning laser microscope (Leica TCS SP2).

### 3.10. Statistical Analysis

The Student’s *t*-test was used to compare the average intensities of WB bands, average viabilities, and average cell counts. One or two asterisks indicate a significant difference, 0.01 < *p* < 0.05 and *p* < 0.01, respectively.

## 4. Conclusions

This study sheds light on the role of Notch1 in human IFE, and demonstrates for the first time the presence of a Notch/survivin axis in keratinocytes, with particular regard to KSC. Given the high expression levels of both Notch and survivin in KSC, markedly declining in TA cells, one can envisage a critical role of the Notch-survivin axis in preserving stemness at the niche level. Indeed, both molecules act as anti-apoptotic factors and regulate cell proliferation ([32,33], present work), providing a mutual cooperation within the niche. It is now widely accepted that while KSC maintain their number [34], proliferation appears to decline with age [35]. Consistently, we report that the Notch/survivin axis stimulates keratinocyte proliferation and markedly decreases with age, suggesting, in agreement with other works [36], that local environment and alteration of the niche modulates skin ageing. KSC are considered to be the origin of skin cancer, while Notch mutation is an early event in cutaneous squamous cell carcinoma (SCC) [37]. Moreover, survivin is implicated in stem cell-derived SCC formation [38]. Because KSC are also responsible for tumor recurrences, targeting the Notch-survivin axis in these cells could result in strong anti-cancer activity.
